# Supplementary material for: Gestational age, birth weight, and perinatal complications in mothers with diabetes and impaired glucose tolerance: Japan Environment and Children’s Study cohort
Source: PLoS One. 2022 Jun 6;17(6):e0269610. doi: 10.1371/journal.pone.0269610 (PMC9170270; doi:10.1371/journal.pone.0269610)
Supplement: S1 Table — (DOCX) [file pone.0269610.s001.docx]

**Relative risks of perinatal outcomes of mothers with type 1 diabetes, type 2 diabetes, gestational diabetes, and impaired glucose tolerance.**

| Relative risk | Type 1 diabetes | Type 2 diabetes | Gestational diabetes | Impaired glucose tolerance | Non-diabetes |
| --- | --- | --- | --- | --- | --- |
| Preterm birth | 2.77^*^ | 2.65* | 1.57* | 1.29* | Reference |
| Caesarean section | 1.79* | 1.98* | 1.40* | 1.19* | Reference |
| Multiple births | ― | 0.80 | 2.12* | 1.35* | Reference |
| Miscarriage | ― | ― | ― | ― | Reference |
| Stillbirth | ― | ― | ― | ― | Reference |
| Any labour complication | 1.04 | 1.49* | 1.59* | 1.22* | Reference |
| Transverse presentation | ― | ― | ― | ― | Reference |
| Breech presentation | 1.45 | 1.59 | 1.59* | 1.08 | Reference |
| Preterm labour | 0.65 | 1.29 | 1.10 | 0.96 | Reference |
| Early rupture of the membranes | 1.71 | 1.37 | 0.69* | 1.07 | Reference |
| Placental abruption | ― | ― | ― | ― | Reference |
| Gestational hypertension | 4.07* | 5.84* | 2.00* | 1.63* | Reference |
| Intrauterine infection | ― | ― | ― | ― | Reference |
| Any neonatal complication | 3.18* | 2.28* | 1.95* | 1.30* | Reference |
| Macrosomia | ― | ― | ― | ― | Reference |
| Jaundice with treatment | 2.04* | 1.99* | 1.02 | 1.12* | Reference |
| Abnormalities | 3.55* | 1.03 | 1.36 | 1.13 | Reference |
| Heart | 2 | 1.19 | 2.44* | 1.54* | Reference |
| Cleft | ― | ― | ― | ― | Reference |
| Umbilical cord hernia | ― | ― | ― | ― | Reference |
| Hypospadias | ― | ― | ― | ― | Reference |
| Chromosomal abnormalities | ― | ― | ― | ― | Reference |

The relative risk was calculated by Poisson regression with adjustment for maternal age and maternal smoking status in the first trimester in pregnancy. The “―” symbol indicates that the analysis was not possible. *p<0.05 compared with the non-diabetes group.

GDM, gestational diabetes.
